# Reevaluating Medication Reduction and Functional Outcomes after Stroke

**DOI:** 10.31662/jmaj.2026-0075

**Published:** 2026-04-24

**Authors:** Jahanzaib Khan

**Affiliations:** 1 Gujranwala Medical College, University of Health Sciences, Lahore, Pakistan

**Keywords:** stroke, medication reduction, functional outcomes, muscle strength, polypharmacy, rehabilitation, patient safety

## To the Editor,

The study “Medication reduction is associated with improved activities of daily living and muscle strength in post-stroke patients with polypharmacy” by Ayaka Matsumoto et al ^(1)^ provides real-world data suggesting an association between medication reduction and improved muscle strength in post-stroke patients with polypharmacy. The topic is clinically important in aging stroke populations; however, several methodological considerations warrant further discussion.

First, although propensity score matching was applied, the retrospective design limits causal inference. Propensity methods balance measured covariates but cannot eliminate bias from unmeasured confounding or incomplete adjustment. In observational clinical research, residual confounding frequently persists despite statistical matching ^(2)^. In stroke rehabilitation settings, factors such as baseline frailty, cognitive status, and rehabilitation intensity may strongly influence both deprescribing decisions and recovery trajectories. Reverse causation, in which patients who are improving are more likely to undergo medication reduction, also remains plausible.

Second, the discordance between improved handgrip strength and reduced skeletal muscle mass index introduces biological uncertainty. While strength and muscle mass are related, they do not change in strict parallel, particularly in older adults, in whom neural adaptation and muscle quality play significant roles ^(3)^. The observed negative association between medication reduction and skeletal muscle mass index warrants further mechanistic clarification, including potential measurement or nutritional factors.

Third, medication reduction was analyzed quantitatively rather than qualitatively. The clinical implications of deprescribing depend fundamentally on which medication classes are withdrawn. Contemporary prescribing frameworks emphasize appropriateness rather than medication count alone, distinguishing between necessary and potentially inappropriate medications ^(4)^. Without class-specific analysis or structured deprescribing criteria, interpretation of the observed associations remains limited.

The study contributes valuable hypothesis-generating data; however, caution is warranted before inferring that medication reduction itself improves functional recovery after stroke. Prospective studies employing structured deprescribing protocols and comprehensive adjustment for patient vulnerability are needed to clarify causal effects on functional recovery.

Sincerely,

Jahanzaib Khan

MBBS, University of Health Sciences, Lahore, Pakistan

jahanzabniazii@gmail.com

## Article addressed

Matsumoto A, Yoshimura Y, Wakabayashi H, et al. Medication reduction is associated with improved activities of daily living and muscle strength in post-stroke patients with polypharmacy. JMA J. 2026;9(1):198-208. https://doi.org/10.31662/jmaj.2025-0264.

## Article Information

### Author Contributions

Jahanzaib Khan conceptualized the letter, drafted the manuscript, critically revised it for intellectual content, and approved the final version for submission.

### Conflicts of Interest

None
